# *LINC00662* enhances cell progression and stemness in breast cancer by MiR-144-3p/*SOX2* axis

**DOI:** 10.1186/s12935-022-02576-0

**Published:** 2022-05-12

**Authors:** Congjing An, Zhigang Hu, Yuehong Li, Pengxin Zhao, Runtian Liu, Qing Zhang, Peiling Zhu, Yanting Li, Ying Wang

**Affiliations:** 1 Department of Breast and Thyroid Surgery, the Second Hospital of Hebei Medical University, Xinhua District, No.215, Heping Xi Road, Shijiazhuang, 050000 Hebei China; 2 Department of Pathology, the Second Hospital of Hebei Medical University, Xinhua District, No.215, Heping Xi Road, Shijiazhuang, 050000 Hebei China

**Keywords:** *LINC00662*, Migration, Invasion, Stemness, miR-144-3p, *SOX2*, Breast cancer

## Abstract

**Background:**

Breast cancer (BC) is one of the most prevalent malignancies among women globally. Emerging evidence indicates that long non-coding RNAs (lncRNAs) are associated with BC carcinogenesis. In the current study, we explored the mechanism by which *LINC00662* regulates BC.

**Methods:**

Quantitative real-time PCR (qRT-PCR) assessed RNA expressions while western blot for protein levels. Kaplan Meier analysis evaluated overall survival (OS). Cytoplasmic/nuclear fractionation, RNA binding protein immunoprecipitation (RIP) and luciferase reporter assays probed into the underlying molecular mechanism of *LINC00662* in BC. Xenograft model was established to explore the influence of *LINC00662* on BC progression in vivo. R square graphs were utilized to represent RNA relationships.

**Results:**

*LINC00662* is overtly overexpressed in BC tissues and cell lines. *LINC00662* knockdown hampers cell proliferation, migration, invasion and stemness. *LINC00662* expression is negatively correlated with OS of BC patients. *LINC00662* up-regulates *SOX2* expression by competitively binding to miR-144-3p, thereby modulating BC cell progression. Xenograft experiments verified that *LINC00662* promotes BC tumor growth and cell stemness in vivo.

**Conclusion:**

*LINC00662* enhances cell proliferation, migration, invasion and stemness in BC by targeting miR-144-3p/*SOX2* axis. The findings in the present study suggested that *LINC00662* could be a potential therapeutic target for BC treatment.

**Graphical Abstract:**

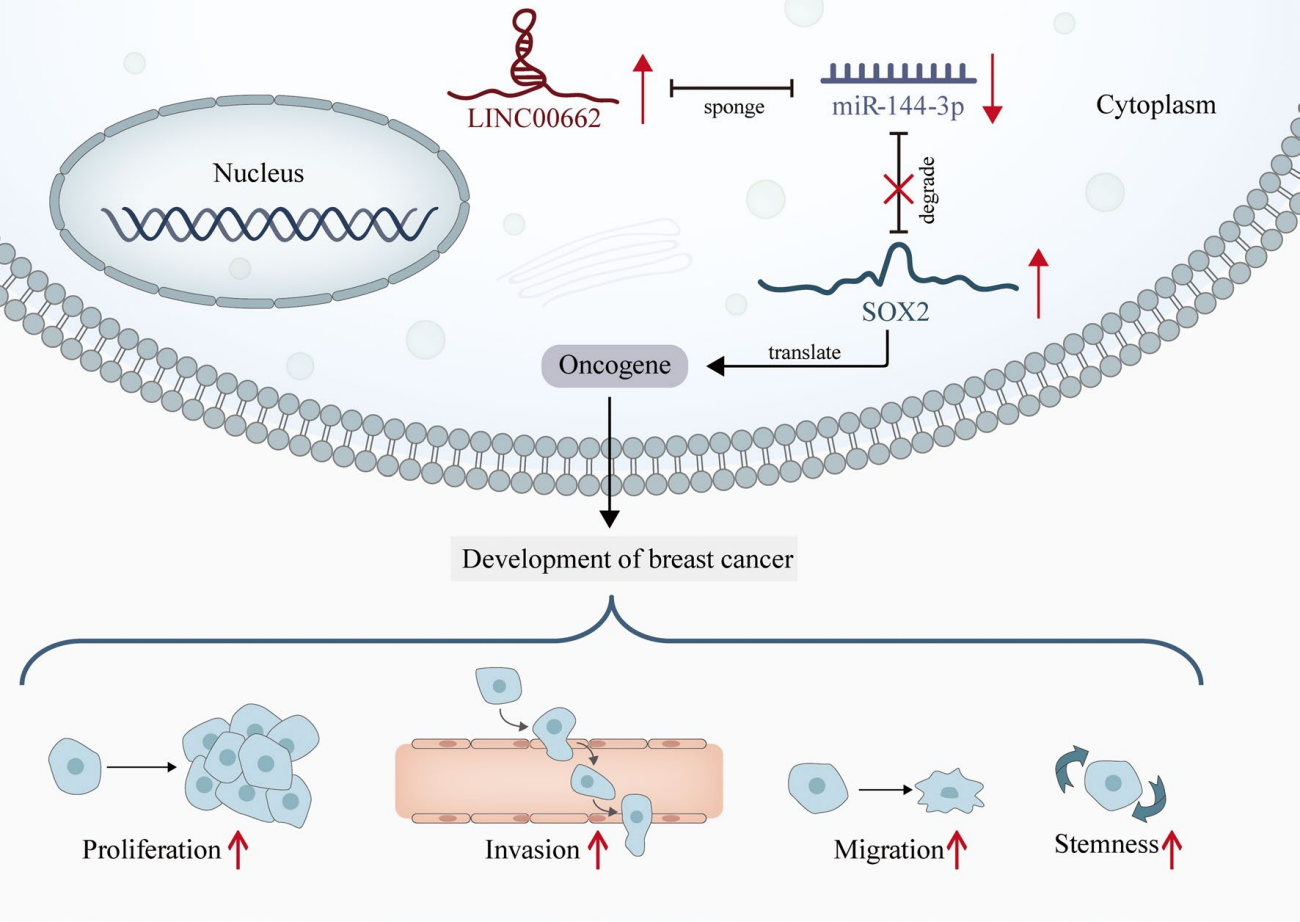

**Supplementary Information:**

The online version contains supplementary material available at 10.1186/s12935-022-02576-0.

## Background

Breast cancer (BC), originating from mammary epithelial tissue, is the most common malignancy among women. Diet-related factors are involved with BC incidence. According to previous study, dietary acid load is directly associated with BC risk (https://scimedjournal.org/index.php/SMJ/article/view/315). In addition, breast malignancy has been reported to be correlated with segmental distribution, clustered-ring enhancement and other factors [1]. Despite the fact that noticeable progress has been made in BC treatment, it is still a devastating disease for female populations [2–4]. One of the reasons for its lethality is due to the shortage of more efficient therapeutic targets [5]. Therefore, understanding the underlying mechanism and identifying novel efficient targets for diagnosis and treatment of BC are thus exigent.

Only 2% of human genomes are protein-coding genes while 98% are non-coding genes. LncRNAs, without protein coding potentials, are a class of non-coding RNAs, featuring no less than 200 nucleotides. Recent studies have reported lncRNAs may function as tumor promoters or tumor suppressors by regulating numerous biological behaviors such as cell proliferation, apoptosis, metastasis and EMT [6–9]. For example, *SNHG5* accelerates BC progression by promoting the proliferation of cancerous cells [10]. *LINC00339* aggravates hepatocellular carcinoma by accelerating cell proliferation and metastasis [11].

According to the previous studies, *LINC00662* has been validated to play an oncogenic role in several cancers, such as oral squamous cell carcinoma and prostate cancer [12, 13]. Furthermore, *LINC00662* facilitates BC cell proliferation and migration by modulating the miR-497-5p/*EglN2* axis [14]. Yet, the mechanism and functions of *LINC00662*/miR-144-3p/*SOX2* regulatory axis in BC was never explored.

Additionally, competing endogenous RNA (ceRNA) mechanism, referring to lncRNA competing with target RNA for miRNA and thus modulating target gene expression, are gaining popularity [15]. The ceRNA mechanism hypothesis provides the researchers with a new insight into research on numerous uncharacterized lncRNAs [16]. As the miRNA in our study, miR-144-3p is implicated in the ceRNA networks along with a variety of lncRNAs in several human cancers. For instance, *TUG1* propels the development of osteosarcoma by targeting miR-144-3p/*EZH2* axis [17–21]. Similarly, *EBLN3P* facilitates the liver cancer progression through miR-144-3p/*DOCK4* axis [22]. Yet the ceRNA network of miR-144-3p in BC still remains unclear.

In the current study, we ascertained that *LINC00662* was overexpressed in BC tissues and cell lines. Furthermore, *LINC00662* knockdown hindered BC cell proliferation, migration, invasion and stemness. MiR-144-3p and *SOX2* were identified as the downstream targets of *LINC00662*. We also found that *LINC00662* propels BC progression in vivo. With the above results, it is hopeful to offer insights into a novel ceRNA mechanism in BC and potential targets for clinical diagnosis and therapeutics for BC.

## Materials and methods

### Specimens

35 paired BC tissues and adjacent non-tumorous tissues were acquired from BC patients in our hospital. The clinicopathological features of patients were shown in Additional file 3: Table S1 and Additional file 4: Table S2. Prior to surgery, no patients had received radiotherapy or chemotherapy. Patients with other kinds of tumors were excluded from this study. The patients put their signatures to the written informed consents prior to sample collection. Subsequent to surgical resection, the excised tissues were snap-frozen in liquid nitrogen and stored at − 80 °C. Kaplan–Meier’s analysis was applied for studying overall survival (OS) with a log-rank test. This study was supported by the ethical committee of our Hospital.

### Cell culture

Normal human mammary epithelial cell line (MCF-10A) and human BC cell lines (MDA-MB-453, MDA-MB-231, MCF-7, and MDA-468) were attained from ATCC (Manassas, VA, USA). MCF-10A cells were kept in DMEM/F12 (D9785, Sigma-Aldrich, St. Louis, MO, USA) with the supplementation of 5% HS (23491-45-4, Sigma-Aldrich), 20 ng/mL epidermal growth factor (EGF; PHG0311, Thermo Fisher Scientific, Rockford, IL, USA), 0.5 μg/mL hydrocortisone (211H-500, Sigma-Aldrich), 1% NEAA (M7145, Sigma-Aldrich), 10 μg/mL insulin (12,643, Sigma-Aldrich) and 1% P/S (PB180120, Procell, Wuhan, China). MCF-7 cells were cultivated in DMEM (D9785, Sigma-Aldrich) with 10% fetal bovine serum (FBS; Gibco, Rockville, MD, USA), 10 µg/mL human insulin and 1 µM 4-hydroxytamoxifen (SML1666, Sigma-Aldrich). MDA-MB-231, MDA-468 and MDA-MB-453 cells were cultivated in ATCC-formulated Leibovitz’s L-15 Medium (11,415,049, Thermo Fisher Scientific) with 10% FBS. All the cells were maintained at 37 °C with 5% CO_2_. The STR reports were provided in Additional file 5.

### Cell transfection

BC cells were subjected to transfection with specific short hairpin RNAs (shRNAs) against *LINC00662* (sh-*LINC00662*#1, sh-*LINC00662*#2, and sh-*LINC00662*#3) and their corresponding NCs, and the pcDNA3.1 vector against *SOX2* or *LINC00662* and the empty vector. The plasmids were synthesized by Genechem (Shanghai, China). Besides, miR-144-3p mimics or miR-144-3p inhibitors and corresponding negative controls (miR-NCs) constructed by GenePharma (Shanghai, China) were co-transfected with sh-*LINC00662* into BC cells. These transfected cells were seeded in the 24-well plate without FBS. Then, transfection was carried out using 1 μL Lipofectamine 3000 (L3000075, Invitrogen, Carlsbad, CA, USA) in 50 μL serum-free medium (Invitrogen) on the basis of supplier’s suggestions.

### Quantitative real-time PCR (qRT-PCR)

QRT-PCR was implemented as per the previous literature [23]. Briefly, Trizol (15596018/15596026, Invitrogen) was used for total RNA extraction from BC cells. Then, extracted RNAs were reverse transcribed to cDNA via PrimeScript™ RT Master Mix (RR036Q, Takara, Tokyo, Japan). Subsequently, qRT-PCR was conducted to investigate gene expression on the LightCycler96 real-time PCR system (Roche, Basel, Switzerland) using the SYBR Green PCR Master mix (4309155, Applied Biosystems, Foster city, CA, USA). 40 cycles of PCR were achieved at 95 °C for 30 s and 57 °C for 30 s. For extension, the temperature was set as 72 °C for 2 min. After the purification, results were calculated in accordance with the 2^−ΔΔCt^ approach with GAPDH or U6 being the control. This assay was implemented in triplicate and each replicate contained three repetitions.

### Cell colony formation assays

Soft agar was applied in assays as previously described [27]. Briefly, transfected cells (500 cells/well) were inoculated into 6-well plates. The plates were subjected to incubation at 37 °C with 5% CO_2_ for two weeks, with culture medium changed at regular intervals. After that, phosphate-buffered saline (PBS; P4417, Sigma-Aldrich) was utilized to wash cells. Then, cells were fixed by 10% formalin (Z2902, Sigma-Aldrich) and stained with 0.5% crystal violet (V5265, Sigma-Aldrich). For the formation efficiency calculation, 5 fields were randomly selected to be counted.

### Transwell assays

Matrigel-uncoated or Matrigel pre-coated transwell inserts (Millipore, Bedford, MA, USA) were employed for evaluation of cell migration or invasion. Transfected cells were inoculated to the upper chambers in medium with no FBS, while 600 μL of 100% FBS was added to the lower chambers. After 12 or 16 h, cells in the upper chambers were slightly wiped using a cotton swab. Migrated or invaded cells into the lower chambers were subjected to fixation with methanol (34,860, Sigma-Aldrich) and staining with crystal violet, followed by observation under an Olympus inverted microscope (Tokyo, Japan, 10 × 10). Lastly, the cells were counted by MShot Image Analysis System and 5 fields per chamber were observed.

### Sphere formation assay

Sphere formation assay was carried out as reported previously [24]. Concisely, transfected MDA-MB-231 or MCF-7 cells were inoculated in ultra-low attachment 6-well plates (Corning, NY, USA; 5 × 10^3^ cells per well) for 10–14 days. Cells were maintained in DMEM/F12 serum-free medium (PM150312A, Procell) added with 5 μg/mL insulin (Sigma-Aldrich), 0.4% bovine serum albumin (BSA; A1933, Sigma-Aldrich), 2% B27 (MAB1285, Sigma-Aldrich), 20 ng/mL basic fibroblast growth factor (bFGF; PHG0369, Thermo Fisher Scientific), and 20 ng/mL EGF (Peprotech, Rocky Hill, NJ, USA). Generated spheres were photographed and then counted using a light microscope (Zeiss, Oberkochen, Germany). Sphere formation efficiency is calculated as (number of spheres/number of inoculated cells) × 100%, using MShot Image Analysis System. Only spheres with diameters greater than 75 μm were counted.

### Western blot

Western blot were carried out in line with standard protocols as previously described [25]. Anti-OCT4 (ab181557), anti-SOX2 (ab97959), anti-Nanog (ab109250) and anti-GAPDH (ab8245) were commercially attained from Abcam (Cambridge, USA). Briefly, total proteins isolated from BC cells were processed with SDS-PAGE for separation and transferred to PVDF membranes, followed by being blocked with 5% skim milk. Afterwards, the membranes were subjected to incubation with primary antibodies overnight 4 °C. The membranes were then washed in TBST, followed by incubation with secondary antibodies for 1 h at room temperature. The blots were then visualized using ECL. GAPDH was applied as the internal control.

### Subcellular fractionation assay

Nuclear and Cytoplasmic Extraction Reagents (78,833, Thermo Fisher Scientific, Waltham, MA, USA) was employed to perform the subcellular fractionation as per the supplier’s protocol. The cells were placed on ice and resuspended in the buffer solution. After 10 min of incubation, the cells were centrifuged for the separation of cytoplasm and nucleus. The RNAs were extracted using Trizol. QRT-PCR was implemented to measure the extracted RNAs. U6 or GAPDH acted as the nuclear or cytoplasmic control.

### RNA binding protein immunoprecipitation (RIP)

RIP assays were carried out as described previously [23]. Briefly, anti-Ago2 antibodies (TS-10X10ML-U, Millipore) or anti-IgG antibodies (MABE-253, Sigma-Aldrich) were incubated with protein A/G agarose beads (78,610, Thermo Fisher Scientific) at 4 °C for the whole night. BC cells were washed in ice-cold PBS, followed by being lysed. Afterwards, cell lysates were incubated with the A/G agarose beads conjugated with anti-Ago2 antibodies or anti-IgG antibodies overnight at 4 °C. When the immunoprecipitation was fully achieved, RNAs were extracted via Trizol from A/G agarose beads and antibodies. Finally, qRT-PCR was applied to measure the relative expression of RNAs (*LINC00662*, miR-144-3p or *SOX2*).

### Cell Counting Kit-8 (CCK-8)

CCK-8 (Dojindo, Kumamoto, Japan) was performed for assessing the proliferative ability of BC cells. The transfected BC cells were planted into 96-well plates, followed by the addition of CCK-8 solution. Next, the cells underwent 24, 48, 72 and 96 h of incubation, followed by addition of 100 μL CCK-8 solution to each well. Afterwards, the incubation lasted for another 4 h. The absorbance (OD) was examined at 450 nm through a microplate reader (Bio-Rad, Hercules, CA, USA).

### Luciferase reporter assay

The sequences of wild type or mutant type *SOX2*/*LINC00662* were sub-cloned into pmirGLO dual-luciferase vectors (Promega, Madison, WI, USA). Then, the luciferase reporter vectors were transfected into BC cells along with miR-144-3p mimics or miR-144-3p mimics + pcDNA3.1/*LINC00662* or miR-NC via Lipofectamine 3000. Firefly and Renilla luciferase activities were analyzed by Dual-luciferase reporter assay system (Promega), normalized to the empty vectors. The signals from luciferase were detected by use of fluorescence microscope (DMI8, Leica, Wetzlar, Germany).

### Xenograft tumor formation assay

Twenty nude mice (6-week-old, female) procured from Shanghai Laboratory Animal Co., Ltd. (SLAC; Shanghai, China) were adopted for the in vivo assay, which was permitted by the Institutional Animal Care and Use Committee of our institution (2021-AE027). The transfected MDA-MB-231 cells were subcutaneously injected into the right flank of mice. Then, the nude mice were put into the euthanasia cage and exposed to carbon dioxide (CO_2_) for 5 min. The CO_2_ flow rate were displaced 10% of the cage volume per minute. Tumor growth was monitored every 4 days and tumor volume was estimated by length and width. 4 weeks post injection, the nude mice were sacrificed. Tumors resected from the nude mice were weighed with an electronic scale.

### Immunohistochemical (IHC) analysis

Ki67 antibodies (ab16667, 1/200, Abcam, Cambridge, MA, USA) and caspase 3 antibodies (ab32351, 1/100, Abcam) were used for immunohistochemical analysis. The tissues from xenograft tumor treated with hydrogen peroxide were incubated with primary antibodies against Ki67 and caspase 3 overnight at 4 °C. Next, secondary antibodies were added to tissues for incubation at 37 °C for 1 h. Subsequently, tissues were stained with DAB and the expression of Ki67 or caspase 3 was measured through a light microscope (Zeiss, Oberkochen, Germany).

### Statistical analysis

All assays were carried out in triplicate. Student’s t-test or one-way ANOVA was utilized for the comparison between groups. R square analysis was applied for analyzing the correlation between *SOX2*, *LINC00662* and miR-144-3p expressions. The statistical analyses in this study were conducted with the application of GraphPad Prism (GraphPad, La Jolla, CA, USA). P < 0.05 was considered as the threshold for statistical significance.

## Results

### *LINC00662* is overexpressed in BC tissues and propels cell proliferation, migration, invasion and stemness and inhibits cell apoptosis

**Fig. 1:**
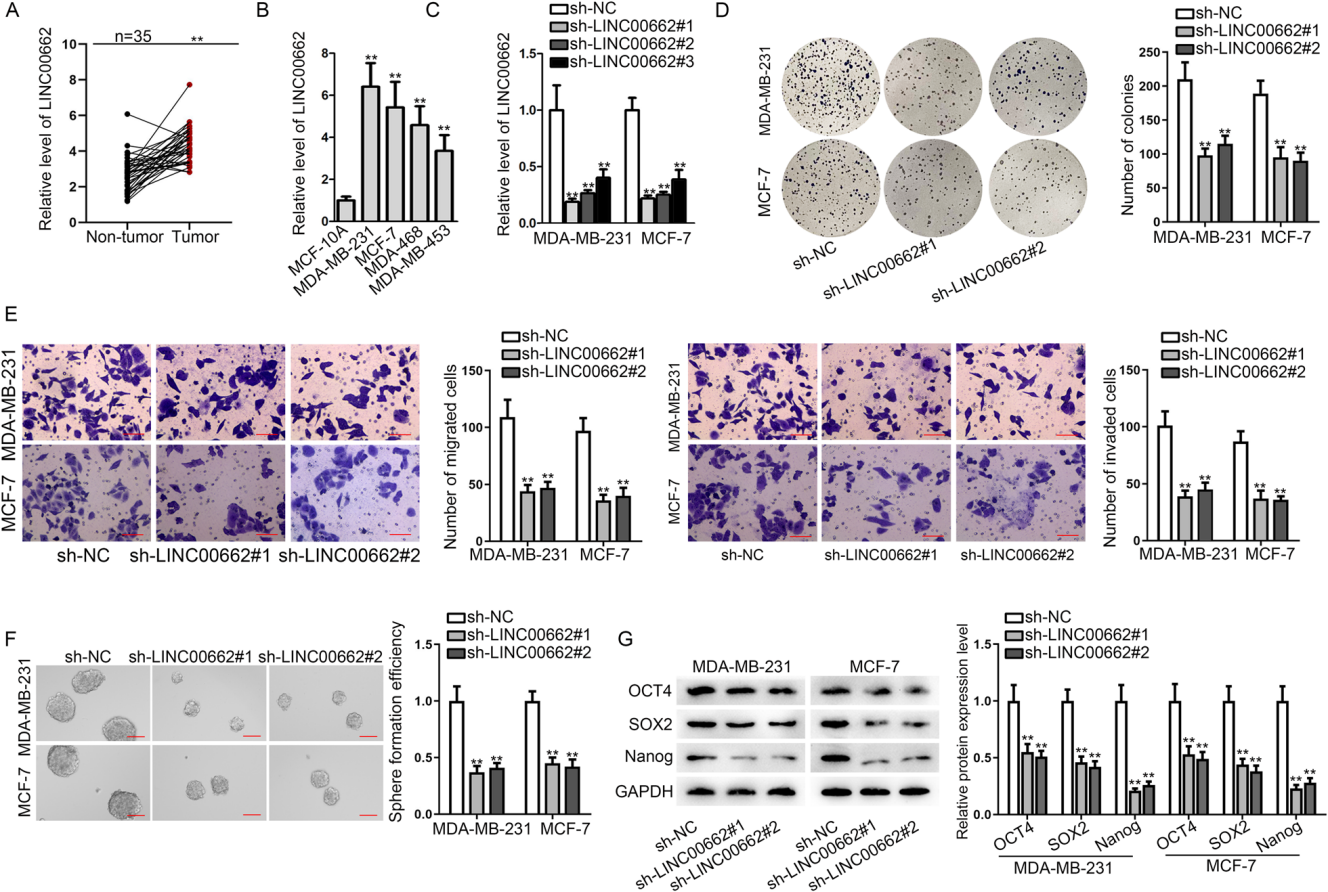
*LINC00662* is overexpressed in BC tissues and promotes cell proliferation, migration, invasion and stemness. **A** QRT-PCR analysis was applied to detect *LINC00662* expression in normal tissues and tumorous tissues. The mean, SD and 95% CI have been provided in Additional file 6: Table S4. **B** QRT-PCR analysis was applied to detect *LINC00662* expression in MCF-10A, MDA-MB-231, MCF-7, MDA-MB-468 and MDA-MB-453. **C** QRT-PCR was employed to examine the efficiency of sh-*LINC00662*#1/2/3. **D** Cell colony formation assay disclosed cell proliferative ability after the silencing of *LINC00662* in MDA-MB-231 and MCF-7 cells. **E** Transwell assays were conducted to investigate cell migratory and invasive ability after knockdown of *LINC00662* in BC cells. **F** Sphere formation assay was applied to detect cell stemness after *LINC00662* knockdown in BC cells. **G** Western blot assay was conducted to examine the levels of stemness-related proteins after *LINC00662* ablation in BC cells. ^**^p < *0.01*

We applied qRT-PCR analysis to evaluate *LINC00662* expression in breast tumor tissues and corresponding adjacent normal tissues. It was uncovered that expression of *LINC00662* was overtly elevated in tumor tissues relative to that in normal tissues (Fig. 1A). 35 patients were split into two groups, one with high expression of *LINC00662*, the other with low expression. As shown in Additional file 1: Fig. S1A, statistical analyses indicated that *LINC00662* expression was negatively linked to OS. We detected *LINC00662* expression respectively in normal breast cell line MCF-10A and cancerous cell lines (MDA-MB-231, MCF-7, MDA-468, and MDA-MB-453) as well, finding *LINC00662* expression was evidently up-regulated in cancerous cell lines than that in normal cell line. Based on the above-mentioned results and the previous study [14], we conjectured that *LINC00662* is an oncogene in BC cells (Fig. 1B). To further study the association between *LINC00662* and biological behaviors of BC cells, we firstly selected two cancerous cell lines MDA-MB-231 and MCF-7 for our further studies due to the prominent expression of *LINC00662* they presented. Next, we transfected sh-*LINC00662*#1/2/3 into MDA-MB-231 and MCF-7 cell lines, finding that *LINC00662* expression was significantly silenced after the transfection. We chose sh-*LINC00662*#1 and sh-*LINC00662*#2 for follow-up experiments, due to the higher efficiency (Fig. 1C). Subsequently, cell colony formation assay was implemented in BC cells to detect cell proliferation. The results showed that cell colonies were significantly decreased after knockdown of *LINC00662*, indicating that *LINC00662* promotes BC cell proliferation (Fig. 1D). Afterwards, western blot was conducted to detect the protein level of apoptosis marker, cleaved caspase 3 in BC cells after *LINC00662* ablation. It was shown that cleaved caspase 3 level was highly increased, indicating that *LINC00662* inhibits BC cell apoptosis (Additional file 1: Fig. S1B). Transwell assays were carried out next to observe migration and invasion capacity in BC cells. As evidenced by the decrease in migrated and invaded cells, cell migratory and invasive abilities were attenuated after knockdown of *LINC00662* (Fig. 1E). Furthermore, we performed sphere formation assay in BC cells to investigate the effects of *LINC00662* on stemness ability. We examined cell sphere numbers after knockdown of *LINC00662* and found the number was decreased sharply, suggesting a decline in sphere formation efficiency (Fig. 1F). Also, we evaluated the expression level of several stemness related proteins, OCT4, *SOX2* and Nanog after knockdown of *LINC00662*, finding the levels of these proteins were all dropped after *LINC00662* knockdown, further confirming *LINC00662* up-regulation promotes stemness of cancerous cells (Fig. 1G). For further verification, we performed gain-of-function experiments in MDA-MB-453 cells using pcDNA3.1/*LINC00662*. It was shown by the results that cell proliferation, migration, invasion and stemness were facilitated and cell apoptosis was inhibited after *LINC00662* overexpression (Additional file 1: Fig. S1C–F). Taken together, *LINC00662* plays an oncogenic role in BC cells.

### *LINC00662* regulates BC cell progression by competitively binding to miR-144-3p

**Fig. 2:**
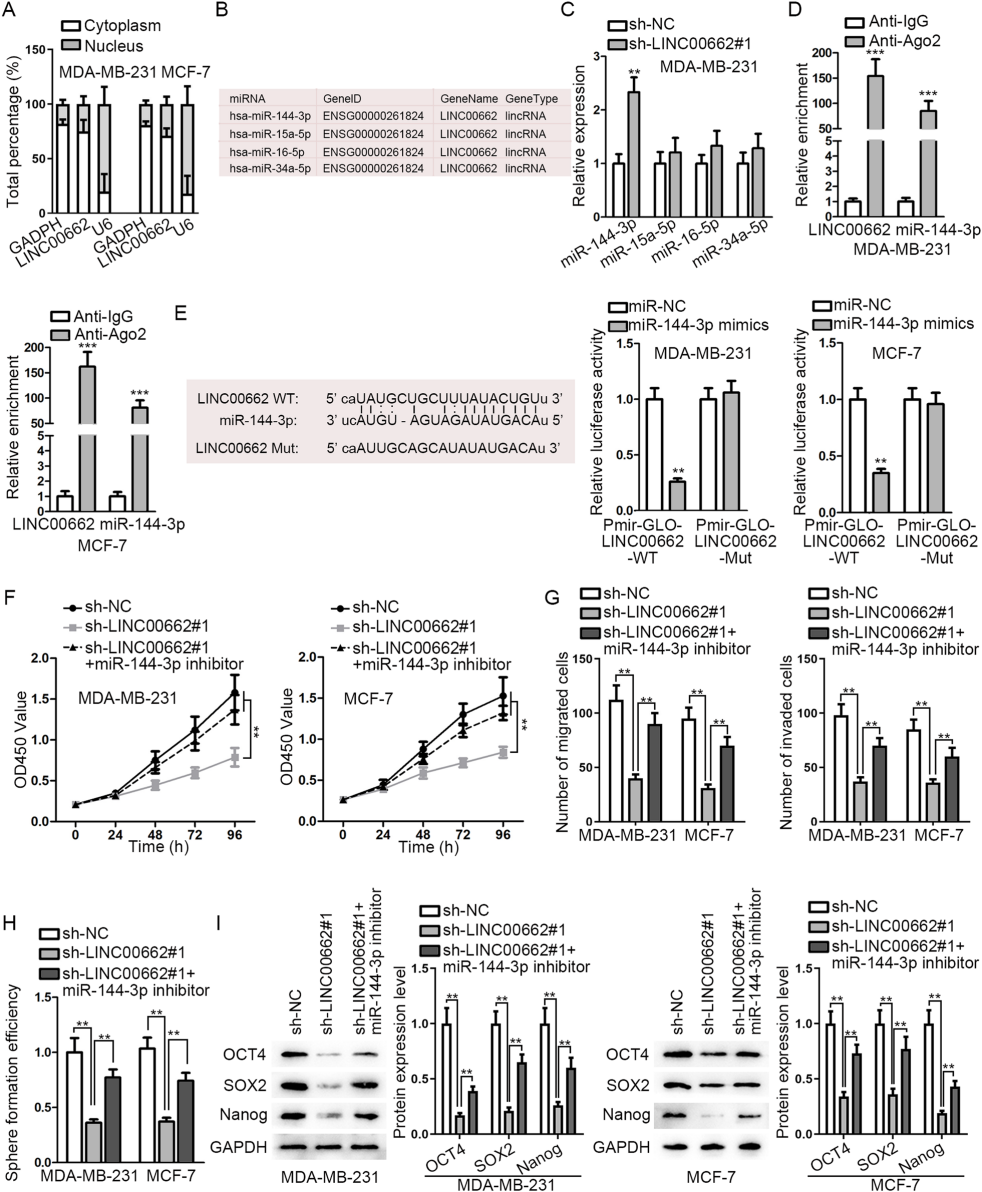
*LINC00662* regulates BC cell progression by competitively binding to miR-144-3p. **A** Cytoplasmic and nuclear fraction RNA analysis was applied to detect *LINC00662* location. **B** Putative miRNAs which share binding sites with *LINC00662* were screened from starBase V2.0. **C** QRT-PCR analysis exhibited relative miRNAs expression after knockdown of *LINC00662*. **D** RIP assay examined relative RNA expression. **E** Binding sites between *LINC00662* wild type (*LINC00662* WT) and miR-144-3p and the base sequence of *LINC00662* mutant type (*LINC00662* Mut) were demonstrated. Luciferase reporter assay verified the putative binding sites between *LINC00662* and miR-144-3p. **F** CCK-8 assay was carried out to evaluate how cell proliferative ability changed after knockdown of *LINC00662* and knockdown of *LINC00662* and miR-144-3p both. **G**–**I** Transwell, sphere formation, and western blot assays were conducted to examine that inhibited cell migration, invasion, stemness ability and stemness-related protein level by knockdown of *LINC00662* were rescued by knockdown of miR-144-3p. ^*^p < 0.05; ^**^p < 0.01

We used lnclocator (http://www.csbio.sjtu.edu.cn/bioinf/lncLocator/) for prediction of the subcellular localization of *LINC00662*. The results of prediction disclosed that *LINC00662* is prominently located in the cytoplasm (Additional file 2: Fig. S2A). As shown in subcellular fractionation assay, the cytoplasmic fraction of *LINC00662* was more enriched than its nuclear fraction, verifying a possibility of ceRNA mechanism (Fig. 2A). To seek the most matched miRNA of *LINC00662*, we retrieved starBase V2.0 (http://starbase.sysu.edu.cn/) and found four miRNAs (miR-144-3p, miR-15a-5p, miR-16-5p, and miR-34a-5p) which share binding sites with *LINC00662* (Fig. 2B). We conducted qRT-PCR in MDA-MB-231 cells to select the one that may be prominently modulated by *LINC00662*. It was unmasked that miR-144-3p expression was strikingly elevated relative to other candidate miRNAs after knockdown of *LINC00662* (Fig. 2C). To further confirm the mechanism between *LINC00662* and miR-144-3p, we performed RIP assay in BC cells. It was disclosed that miR-144-3p and *LINC00662* expressions were enriched in Anti-Ago2 group, confirming that miR-144-3p and *LINC00662* coexists in RNA-induced silencing complex (Fig. 2D). The binding sites between *LINC00662* and miR-144-3p and the base sequence of *LINC00662* mutant type were predicted by starBase V2.0. It was disclosed by luciferase reporter assay that the luciferase activity of WT group was significantly attenuated after miR-144-3p overexpression while that of Mut group had no marked change, verifying that *LINC00662* binds with miR-144-3p (Fig. 2E). Next, a series of rescue experiments were conducted in BC cells transfected with sh-NC, sh-*LINC00662*#1 or sh-*LINC00662*#1 + miR-144-3p inhibitor. First, CCK-8 results demonstrated that cell proliferation was decreased after knockdown of *LINC00662* and was then resumed after co-transfection with miR-144-3p inhibitor (Fig. 2F). As exhibited in Transwell assays, cell migratory and invasive capacities were impaired after knockdown of *LINC00662* and then reversed after co-transfection with miR-144-3p inhibitor (Fig. 2G). The results of western blot showed that cleaved caspase 3 level was increased by *LINC00662* depletion and then counteracted by miR-144-3p inhibition (Additional file 2: Fig. S2B). In addition, sphere formation efficiency which was declined after *LINC00662* knockdown, was then reversed by miR-144-3p knockdown (Fig. 2H). Furthermore, we performed western blot to detect stemness-related protein level and found levels of OCT4, *SOX2* and Nanog shared the same changes as the above results (Fig. 2I). Taken together, *LINC00662* regulates BC cell progression by competitively binding to miR-144-3p.

### *LINC00662 *targets miR-144-3p/*SOX2* axis to modulate BC cell progression

**Fig. 3:**
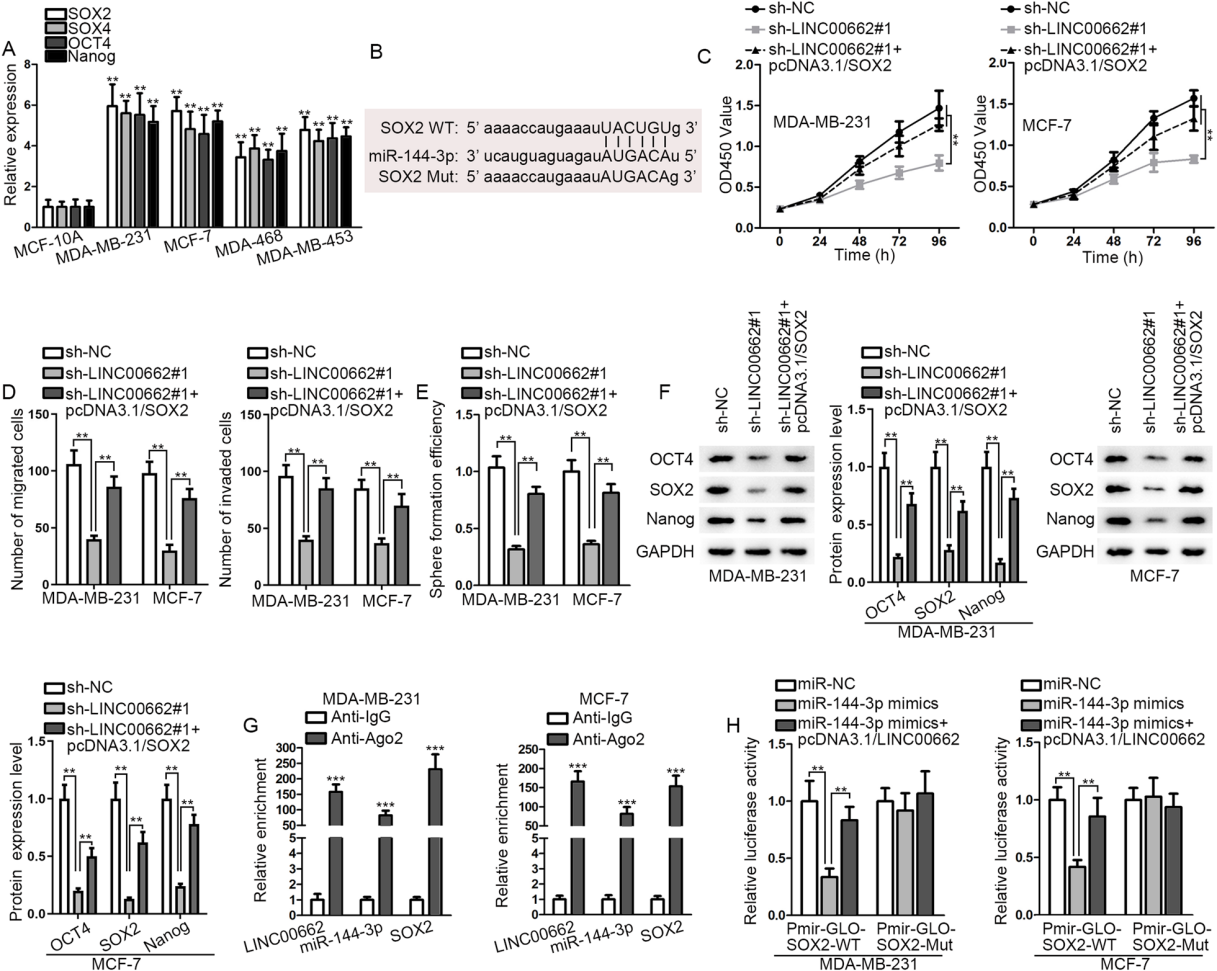
*LINC00662* targets miR-144-3p/*SOX2* axis to modulate BC cell progression. **A** QRT-PCR analysis was employed to investigate the expression of stemness-related genes in MCF-10A, MDA-MB-231, MCF-7, MDA-MB-468 and MDA-MB-453 cells. **B** Binding sites between *SOX2* wild type (*SOX2* WT) and miR-144-3p and the base sequence of *SOX2* mutant type (*SOX2* Mut) were demonstrated. **C** CCK-8 assay evaluated cell proliferative ability after the down-regulation of *LINC00662* or knockdown of *LINC00662* and overexpression of *SOX2*. **D**–**F** Transwell, sphere formation, and western blot assays were conducted to examine cell migration, invasion, stemness ability and stemness-related protein level after the down-regulation of *LINC00662* or knockdown of *LINC00662* and overexpression of *SOX2*. **G** RIP assay evaluated the enrichments of *LINC00662*, miR-144-3p, *SOX2* in RISC in BC cells. **H** Luciferase reporter assay was carried out to verify the ceRNA network among *LINC00662*, miR-144-3p and *SOX2*. ^**^ *p* < 0.01; ^***^ *p* < 0.001

Next, we probed into the downstream mRNA of miR-144-3p. Previous studies verified that *SOX2*, *SOX4*, *OCT4*, and *Nanog* are involved in cell migration, invasion and stemness [26, 27]. Thus, we conjectured that miR-144-3p may mediate cell migration, invasion and stemness via targeting the above genes. Subsequently, we detected the expressions of these genes in MCF-10A and BC cells using qRT-PCR, finding that their expression all rose significantly after knockdown of *LINC00662* (Fig. 3A). According to the prediction by starBase V2.0, only *SOX2* was found sharing binding sites with miR-144-3p (Fig. 3B). Moreover, *SOX2* expression level in BC patients was claimed to be positively related to OS [28, 29]. Thus, *SOX2* was supposed as the downstream target of miR-144-3p. Subsequently, several functional rescue assays were conducted in BC cells after transfection of sh-NC, sh-*LINC00662*#1 or sh-*LINC00662*#1 + pcDNA3.1/*SOX2*. It was displayed by CCK-8 and Transwell assays that cell proliferative, migratory and invasive abilities were suppressed by knockdown of *LINC00662*, and was then resumed by overexpression of *SOX2* (Fig. 3C, D). As shown in Additional file 2: Fig. S2C, cleaved caspase 3 level was increased by *LINC00662* knockdown and then counteracted by *SOX2* overexpression. Similarly, cell stemness ability and stemness-related protein level were both decreased markedly after the silencing of *LINC00662* and recovered after co-transfection with pcDNA3.1/*SOX2* (Fig. 3E, F). Moreover, RIP and luciferase reporter assays were carried out to further confirm the regulatory mechanism among *LINC00662*, miR-144-3p and *SOX2*. The results of RIP suggested that *SOX2*, *LINC00662* and miR-144-3p co-exists in the RISC (Fig. 3G). It was unmasked by luciferase reporter assay that luciferase activity reduced by miR-144-3p mimics was reversed by *LINC00662* (Fig. 3H). Figure 3G, H confirmed the post-transcriptional regulation character and ceRNA mechanism among these three. To sum up, *LINC00662* targets miR-144-3p/*SOX2* axis to modulate BC cell progression.

### Relative expressions of *LINC00662* and *SOX2* are negatively related to miR-144-3p expression that is inhibited in BC tissues

**Fig. 4:**
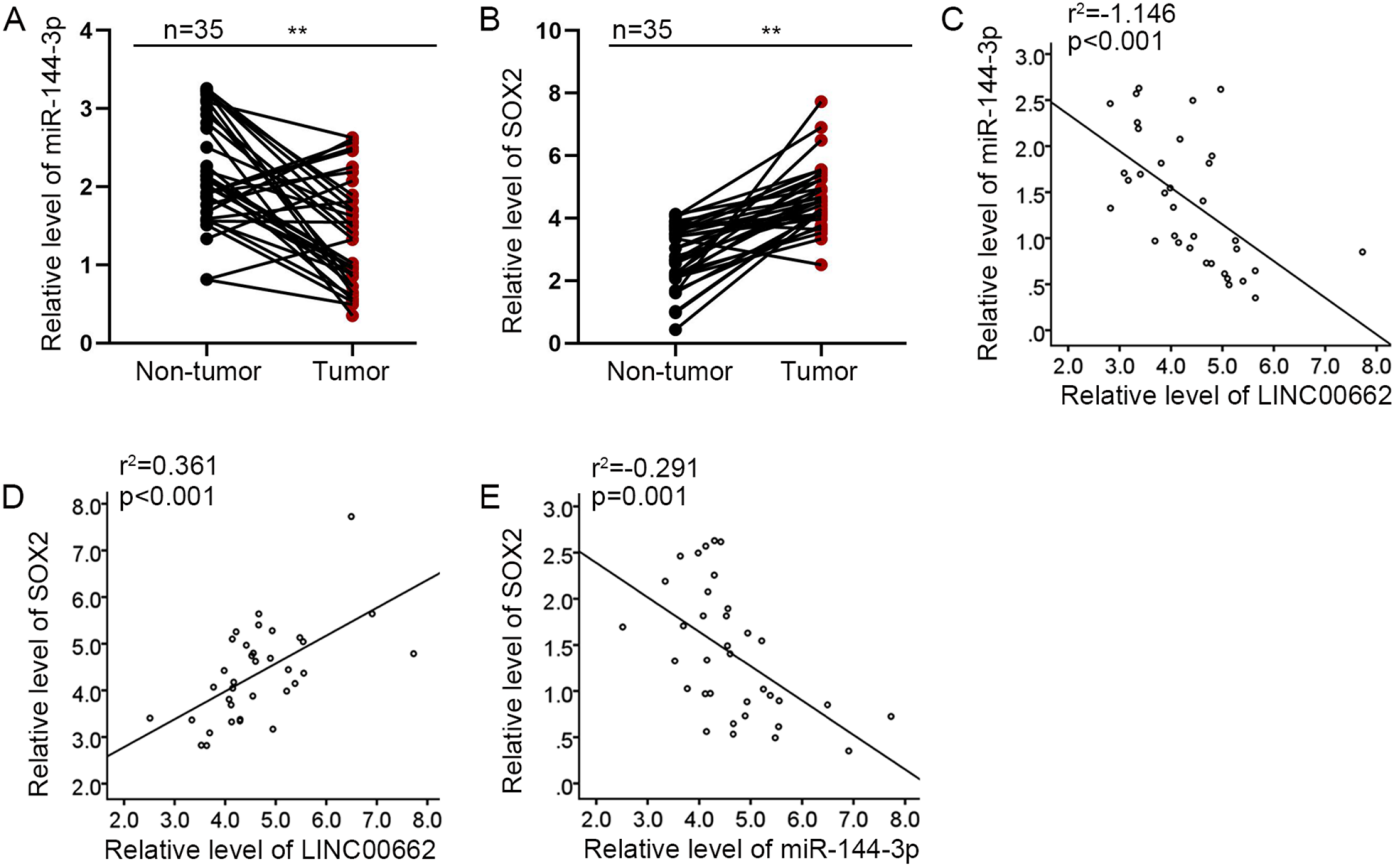
Relative expressions of *LINC00662* and *SOX2* are negatively related to miR-144-3p expression that is inhibited in BC tissues. **A**, **B** QRT-PCR analysis was applied to detect miR-144-3p and *SOX2* expressions in normal tissues and tumorous tissues. The mean, SD and 95% CI have been provided in Additional file 6: Table S4. **C**–**E** Correlation analysis using R square graphs validated the relationship between *LINC00662*, miR-144-3p and *SOX2*. ^**^ *p* < 0.01

It was detected through qRT-PCR that miR-144-3p was notably overexpressed in tumor tissues compared with that in normal tissues while *SOX2* expression was restrained in BC tissues (Fig. 4A, B). Subsequently, correlation analysis using R square graphs indicated that *LINC00662* expression is negatively associated with miR-144-3p expression and is positively linked to *SOX2* expression; and *SOX2* expression is negatively related to miR-144-3p expression in BC tissues (Fig. 4C−E). Taken together, relative expressions of *LINC00662* and *SOX2* are negatively linked to miR-144-3p expression that is inhibited in BC tissues.

### *LINC00662* propels BC tumor growth and cell stemness

**Fig. 5:**
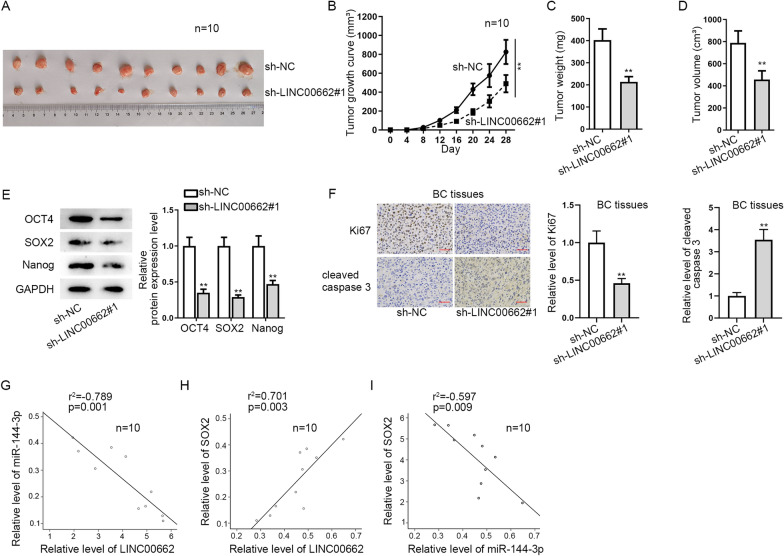
*LINC00662* propels BC tumor growth and cell stemness. **A** Ten nude mice transplanted with subcutaneous xenografts were chosen as the vivo specimens. **B** Tumor growth curve was plotted to present tumor growth along with time. **C**, **D** Tumor weight and volume change was detected after knockdown of *LINC00662*. **E** Expression levels of stemness-related proteins were uncovered through western blot assay in BC tissues excised from mice. **F** Ki67 and cleaved caspase 3 were detected through IHC to evaluate the proliferation and apoptosis of BC tissues. **G**–**I** Correlation analysis using R square graphs confirmed the correlation between *LINC00662*, miR-144-3p and *SOX2 *in vivo. ^**^ *p* < 0.01

Ten nude mice transplanted with subcutaneous sarcoma were chosen as the vivo specimens. Tumors were monitored through the whole assay and changes were observed after the transfection of sh-*LINC00662* into MDA-MB-231 cell line (Fig. 5A). Compared with sh-NC group, an obvious decrease of tumor growth was observed (Fig. 5B). Tumor weight and volume were showed with a significant decrease after knockdown of *LINC00662* as well (Fig. 5C, D). Moreover, the levels of stemness-related proteins were decreased as well after silencing *LINC00662* (Fig. 5E). In addition, IHC staining was used to detect the levels of proliferation marker Ki67 and cleaved caspase 3 to respectively analyze the proliferation and apoptosis of BC tissues excised from nude mice. The result showed that proliferation was sharply shaved while the apoptosis was overtly promoted in sh-*LINC00662* group compared with the control group (Fig. 5F). Correlation analysis using R square graphs were utilized to detect the relationship between *LINC00662*, miR-144-3p and *SOX2*. The graphs showed a negative relationship between *LINC00662* and miR-144-3p, a positive relationship between *LINC00662* and *SOX2* and a negative relationship between miR-144-3p and *SOX2 *in vivo (Fig. 5G−I). So far, ceRNA mode of *LINC00662*/miR-144-3p/*SOX2* axis and its influence on BC progression is clear as shown in Graphical abstract. Thus, we may conclude *LINC00662* promotes migration, invasion and stemness in BC by targeting miR-144-3p/*SOX2* axis, in vitro and vivo.

## Discussion

There has been wide consensus that lncRNAs are profoundly involved in multiple biological processes [30]. LncRNAs have been found to be implicated in different cancers, encompassing BC [2]. For instance, *LINC01287* aggravates BC by Wnt/β-catenin [31], and *UASR1* promotes BC by targeting AKT/mTOR axis [32]. *LINC00662* was proved to serve as an oncogene in several cancers. For instance, *LINC00662* promotes lung cancer [33]. *LINC00662* promotes gastric cancer by targeting Hippo-YAP1 axis [34]. *LINC00662* was also found to accelerate prostate cancer, oral squamous cell carcinoma, colorectal cancer progression [35, 36]. In this present study, we found the overexpression of *LINC00662* in BC tissues and cells. Moreover, through the functional experiments, we verified that *LINC00662* serves as an oncogene to promote BC progression. Previous study has been conducted to indicate that *LINC00662* promotes the development of BC by targeting miR-497-5p/*EglN2* axis [14]. However, the lack of in vivo experiments and analysis of clinical samples undermined the stringency of this study. Our current study explored *LINC00662* expression in BC tissues and verified its regulation in vivo. It has been reported that *LINC00662* is mainly distributed in the cytoplasm of hepatocellular carcinoma cells [37]. In line with the previous study, the prediction of bioinformatics showed that *LINC00662* is prominently distributed in the cytoplasm. Next, cytoplasmic and nuclear fraction RNA analysis confirmed the location of *LINC00662*. This revealed that *LINC00662* was likely to function as a ceRNA, due to the fact that it was mainly located in cytoplasm. According to the literature review, some miRNAs, including miR-34a [38], miR-145 [39], miR-340-5p [40] and miR-497-5p [41], have been reported to interact with *LINC00662*. To select a miRNA that could bind with *LINC00662* in BC, we used bioinformatics and experiments to prove the binding of miR-144-3p to *LINC00662* in BC cells. In accordance with literature review, miR-144-3p is implicated in ceRNA network in liver cancer [22]. In the present study, rescue assays were carried out to confirm that *LINC00662* regulates BC cell progression via competitively binding to miR-144-3p.

To confirm the target gene of miR-144-3p, we selected four reported stemness-related genes. As indicated by bioinformatics analysis, only *SOX2* shared binding sites with miR-144-3p. Thus, *SOX2* was selected as the potential target gene. According to the literature review, we found that *SOX2* is related to ceRNA network in various diseases, encompassing triple negative breast cancer [42] and osteoarthritis [43]. In our study, rescue experiments were conducted to detect biological function change after knockdown of *LINC00662* and co-transfection of pcDNA3.1/*SOX2* into BC cells. Furthermore, RIP assay and luciferase reporter assay further confirmed the ceRNA mechanism among *LINC00662*, miR-144-3p and *SOX2*. In other words, *LINC00662* modulates *SOX2* expression by sequestering miR-144-3p in vitro.

## Conclusion

Taken together, our results suggested that *LINC00662* promotes BC cell proliferation, migration, invasion and stemness and inhibited BC cell apoptosis by activating miR-144-3p/*SOX2* axis. Furthermore, *LINC00662* propels BC tumor growth and cell stemness in vivo. The present study firstly investigated the role of *LINC00662*/miR-144-3p/*SOX2* regulatory axis in BC cells. Furthermore, we firstly analyzed *LINC00662* expression in clinical samples and associated its expression with OS of BC patients. The findings in this study highlight the possibility that *LINC00662* might be a therapeutic target for BC treatment. However, due to the limitation of fund and manpower, we only probed into the ceRNA mode in BC. In the future, we will explore whether *LINC00662* can regulate BC progression through other mechanisms.

## Supplementary Information

**Additional file 1: Figure S1.** A. OS result was analyzed through Kaplan–Meier to examine the effect of *LINC00662* expression on BC patient survival and clinicopathological features of BC patients were analyzed. B. Cleaved caspase 3 protein level was disclosed by western blot after *LINC00662* depletion in MDA-MB-231 and MCF-7 cells. C. Cell colony formation assay was implemented to evaluate cell proliferation after *LINC00662* overexpression in MDA-MB-453 cells. D. The protein level of cleaved caspase 3 was detected after *LINC00662* overexpression in MDA-MB-453 cells according to western blot. E. Transwell assays were implemented to assess cell migratory and invasive ability after up-regulation of *LINC00662* expression in MDA-MB-453 cells. F. Sphere formation assay was applied to evaluate cell stemness after up-regulation of *LINC00662* expression in MDA-MB-453 cells. ^**^ *p* < *0.01.*

**Additional file 2: Figure S2.** A. The subcellular location of *LINC00662* was attatined based on the prediction by lnclocator (http://www.csbio.sjtu.edu.cn/bioinf/lncLocator/). B. Western blot detected the protein level of cleaved caspase 3 after transfection of sh-NC, sh-*LINC00662* or sh-*LINC00662* + miR-144-3p inhibitor. C. Western blot investigated cleaved caspase 3 protein level after transfection of sh-NC, sh-*LINC00662* or sh-*LINC00662* + pcDNA3.1/*SOX2*. ^**^ *p* < *0.01.*

**Additional file 3: Table S1.** Relationship between *LINC00662* expression and clinical features of breast cancer patients (n = 35).

**Additional file 4: Table S2.** Multivariate analysis of prognostic parameters in breast cancer patients by Cox regression analysis was shown.

**Additional file 5: Table S3.** The transfection efficiency of Figs. 1C, 2F, 3C and the knockdown efficiency of Fig. 1C were shown.

**Additional file 6: Table S4.** The Mean, Standard Deviation and Confidence Interval (CI) in 95% significance level of Figs. 1A, 4A and B. VAR00001 and VAR00002 refer to tumor and non-tumor tissues respectively.

## Data Availability

Not applicable.

## Abbreviations

bFGF: Basic fibroblast growth factor
BSA: Bovine serum albumin
CCK8: Cell Counting Kit 8
ceRNA: Competitive endogenous RNA
DMEM: Dulbecco’s modified eagle medium
EGF: Epidermal growth factor
EMT: Epithelial-mesenchymal transition
IHC: Immunohistochemistry
lncRNA: Long non-coding RNA
PBS: Phosphate buffer saline
qRT-PCR: Quantitative real-time PCR
RIP: RNA binding protein immunoprecipitation
shRNA: Short hairpin RNA
