# Supplementary figures and images for: LINC00662 enhances cell progression and stemness in breast cancer by MiR-144-3p/SOX2 axis
Source: Cancer Cell Int. 2022 May 12;22:184. doi: 10.1186/s12935-022-02576-0 (PMC9097442; doi:10.1186/s12935-022-02576-0)

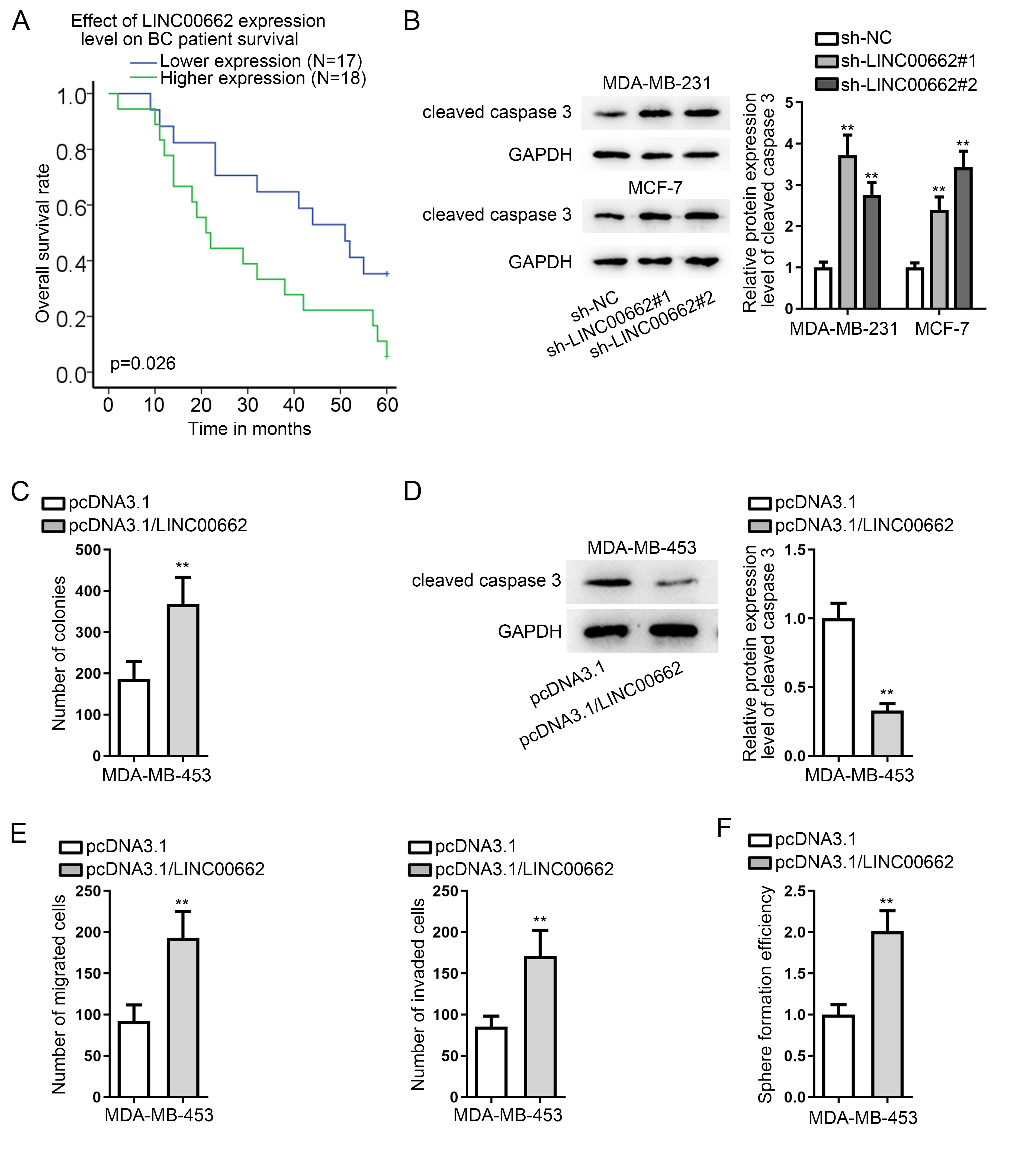

Supplement: Supplementary file 1 — Additional file 1: Figure S1. A. OS result was analyzed through Kaplan–Meier to examine the effect of LINC00662 expression on BC patient survival and clinicopathological features of BC patients were analyzed. B. Cleaved caspase 3 protein level was disclosed by western blot after LINC00662 depletion in MDA-MB-231 and MCF-7 cells. C. Cell colony formation assay was implemented to evaluate cell proliferation after LINC00662 overexpression in MDA-MB-453 cells. D. The protein level of cleaved caspase 3 was detected after LINC00662 overexpression in MDA-MB-453 cells according to western blot. E. Transwell assays were implemented to assess cell migratory and invasive ability after up-regulation of LINC00662 expression in MDA-MB-453 cells. F. Sphere formation assay was applied to evaluate cell stemness after up-regulation of LINC00662 expression in MDA-MB-453 cells. ** p < 0.01. [file 12935_2022_2576_MOESM1_ESM.tif]

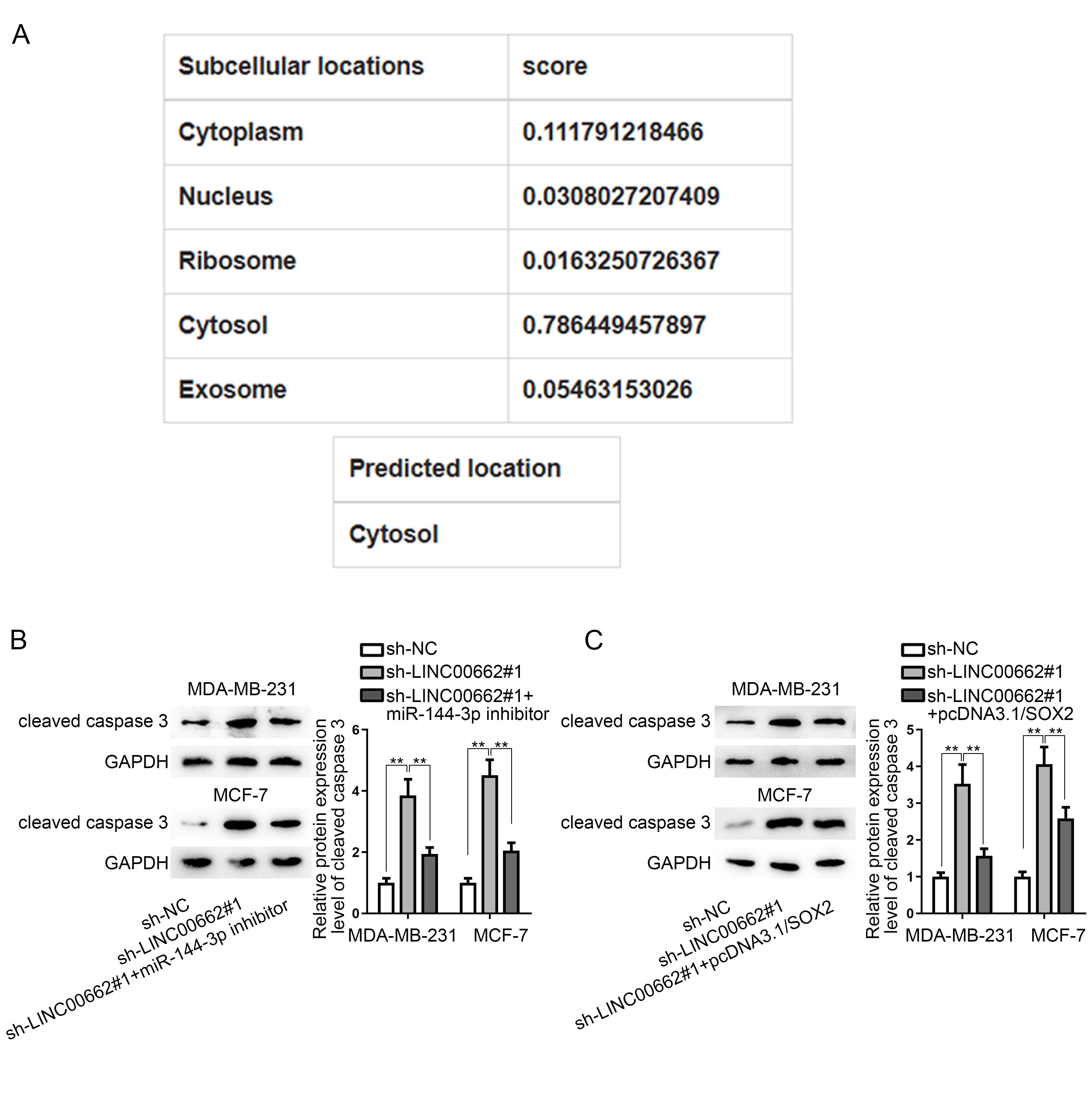

Supplement: Supplementary file 2 — Additional file 2: Figure S2. A. The subcellular location of LINC00662 was attatined based on the prediction by lnclocator (http://www.csbio.sjtu.edu.cn/bioinf/lncLocator/). B. Western blot detected the protein level of cleaved caspase 3 after transfection of sh-NC, sh-LINC00662 or sh-LINC00662 + miR-144-3p inhibitor. C. Western blot investigated cleaved caspase 3 protein level after transfection of sh-NC, sh-LINC00662 or sh-LINC00662 + pcDNA3.1/SOX2. ** p < 0.01. [file 12935_2022_2576_MOESM2_ESM.tif]
