# Supplementary material for: LINC00662 enhances cell progression and stemness in breast cancer by MiR-144-3p/SOX2 axis
Source: Cancer Cell Int. 2022 May 12;22:184. doi: 10.1186/s12935-022-02576-0 (PMC9097442; doi:10.1186/s12935-022-02576-0)
Supplement: Supplementary file 3 — Additional file 3: Table S1. Relationship between LINC00662 expression and clinical features of breast cancer patients (n = 35). [file 12935_2022_2576_MOESM3_ESM.docx]

**Correlation between LINC00662 expression and clinical features of breast cancer patients (n = 35)**

| **Variable** | **Groups** | **LINC00662 expression-Low** | **LINC00662 expression-High** | **P Value** |
| --- | --- | --- | --- | --- |
| **Age** | **<60** | **8** | **5** | **0.305267** |
|  | **>=60** | **9** | **13** |  |
| **Tumor Size** | **<3cm** | **12** | **5** | **0.018394^*^** |
|  | **>=3cm** | **5** | **13** |  |
| **TNM** | **I/II** | **11** | **1** | **0.000297^***^** |
|  | **III/IV** | **6** | **17** |  |
| **LNM** | **Negative** | **13** | **8** | **0.085802** |
|  | **Positive** | **4** | **10** |  |

**TNM=Tumor Node Metastasis, LNM=Lymph Node Metastasis. *p<0.05, **p<0.01 indicated data are statistially sigificant.**
