# Supplementary material for: LINC00662 enhances cell progression and stemness in breast cancer by MiR-144-3p/SOX2 axis
Source: Cancer Cell Int. 2022 May 12;22:184. doi: 10.1186/s12935-022-02576-0 (PMC9097442; doi:10.1186/s12935-022-02576-0)
Supplement: Supplementary file 4 — Additional file 4: Table S2. Multivariate analysis of prognostic parameters in breast cancer patients by Cox regression analysis was shown. [file 12935_2022_2576_MOESM4_ESM.docx]

**Multivariate analysis of prognostic parameters in patients with breast cancer by Cox regression analysis**

| **Parameters** | **Group** | **HR** | **CI (95%)** | **P Value** |
| --- | --- | --- | --- | --- |
| **Age** | **<60** | **0.322** | **0.081-1.275** | **0.106** |
|  | **>=60** |  |  |  |
| **Tumor Size** | **<3cm** | **0.274** | **0.061-1.223** | **0.090** |
|  | **>=3cm** |  |  |  |
| **TNM** | **I/II** | **3.755** | **0.381-37.047** | **0.257** |
|  | **III/IV** |  |  |  |
| **LNM** | **Negative** | **1.128** | **0.28-4.539** | **0.866** |
|  | **Positive** |  |  |  |
| **LINC00662**  **expression** | **Low** | **8.614** | **1.191-62.312** | **0.033^*^** |
|  | **High** |  |  |  |

**TNM=Tumor Node Metastasis, LNM=Lymph Node Metastasis. *p<0.05 indicated data are statistially sigificant.**
