# Supplementary material for: LINC00662 enhances cell progression and stemness in breast cancer by MiR-144-3p/SOX2 axis
Source: Cancer Cell Int. 2022 May 12;22:184. doi: 10.1186/s12935-022-02576-0 (PMC9097442; doi:10.1186/s12935-022-02576-0)
Supplement: Supplementary file 5 — Additional file 5: Table S3. The transfection efficiency of Figs. 1C, 2F, 3C and the knockdown efficiency of Fig. 1C were shown. [file 12935_2022_2576_MOESM5_ESM.docx]

| **Figure** | **Cell** |  | **Transfection efficiency（%）** |
| --- | --- | --- | --- |
| 1C | MDA-MB-231 | sh-NC | 78.21 |
|  |  | sh-LINC00662 | 85.37 |
|  | MCF-7 | sh-NC | 87.65 |
|  |  | sh-LINC00662 | 82.92 |
| 2F | MDA-MB-231 | sh-NC | 77.25 |
|  |  | sh-LINC00662 | 80.68 |
|  |  | sh-LINC00662+ miR-144-3p inhibitor | 74.89 |
|  | MCF-7 | sh-NC | 79.24 |
|  |  | sh-LINC00662 | 81.85 |
|  |  | sh-LINC00662+ miR-144-3p inhibitor | 82.14 |
| 3C | MDA-MB-231 | sh-NC | 80.55 |
|  |  | sh-LINC00662 | 85.67 |
|  |  | sh-LINC00662+ pcDNA3.1/SOX2 | 79.22 |
|  | MCF-7 | sh-NC | 82.41 |
|  |  | sh-LINC00662 | 79.52 |
|  |  | sh-LINC00662+ pcDNA3.1/SOX2 | 86.72 |

| **Figure** | **Cell** |  | **Knockdown efficiency（%）** |
| --- | --- | --- | --- |
| 1C | MDA-MB-231 | sh-NC | 72.83 |
|  |  | sh-LINC00662#1 | 79.81 |
|  |  | sh-LINC00662#2 | 83.49 |
|  |  | sh-LINC00662#3 | 80.49 |
|  | MCF-7 | sh-NC | 76.36 |
|  |  | sh-LINC00662#1 | 79.11 |
|  |  | sh-LINC00662#2 | 74.56 |
|  |  | sh-LINC00662#3 | 80.95 |
