# Supplementary material for: LINC00662 enhances cell progression and stemness in breast cancer by MiR-144-3p/SOX2 axis
Source: Cancer Cell Int. 2022 May 12;22:184. doi: 10.1186/s12935-022-02576-0 (PMC9097442; doi:10.1186/s12935-022-02576-0)
Supplement: Supplementary file 6 — Additional file 6: Table S4. The Mean, Standard Deviation and Confidence Interval (CI) in 95% significance level of Figs. 1A, 4A and B. VAR00001 and VAR00002 refer to tumor and non-tumor tissues respectively. [file 12935_2022_2576_MOESM6_ESM.docx]

| Figure | Genes |  | Mean | SD | 95% CI |
| --- | --- | --- | --- | --- | --- |
| 1A | LINC00662 | VAR00001 | 2.72 | 0.9913 | Lower: 2.3795 Upper: 3.0605 |
|  |  | VAR00002 | 4.3588 | 1.00234 | Lower: 4.0145 Upper: 4.7032 |
| 4A | miR-144-3p | VAR00001 | 2.195 | 0.69602 | Lower: 1.9560 Upper: 2.4341 |
|  |  | VAR00002 | 1.4051 | 0.69669 | Lower: 1.1658 Upper: 1.6445 |
| 4B | SOX2 | VAR00001 | 2.8209 | 0.99166 | Lower: 2.4802 Upper: 3.1615 |
|  |  | VAR00002 | 4.6381 | 1.00851 | Lower: 4.2917 Upper: 4.9846 |

The Mean, Standard Deviation and Confidence Interval (CI) in 95% significance level of Figure 1A, 4A and 4B.
